# Cognitive Outcomes Following Physical Activity Interventions Incorporating Co-Design: A Systematic Review of Randomised Controlled Trials

**DOI:** 10.3390/life16101714

**Published:** 2026-10-14

**Authors:** Ashley Stewart, Thilini D. W. H. Pathiranage, Dean E. Mills, Tracy Kolbe-Alexander, Edward S. Bliss

**Affiliations:** 1 School of Health, Psychological and Medical Sciences, University of Southern Queensland, Ipswich, QLD 4305, Australiathilini.herathpathiranage@unisq.edu.au (T.D.W.H.P.); dean.mills@unisq.edu.au (D.E.M.); tracy.kolbe-alexander@unisq.edu.au (T.K.-A.); 2 Institute of Health Research, University of Southern Queensland, Ipswich, QLD 4305, Australia; 3 UCT Research Centre for Health Through Physical Activity, Lifestyle and Sport (HPALS), Division of Research Unit for Exercise Science and Sports Medicine, University of Cape Town, Newlands, Cape Town 7700, South Africa; 4 Manna Institute, Institute of Health Research, University of Southern Queensland, Ipswich, QLD 4305, Australia; 5 Rural Clinical School, Medical School, Faculty of Health, Medicine and Behavioural Sciences, University of Queensland, Toowoomba, QLD 4350, Australia

**Keywords:** co-design, physical activity, exercise, cognition, cerebrovascular circulation, ageing

## Abstract

Dementia and cerebrovascular dysfunction are growing public health challenges. Although physical activity can improve brain health, real-world adherence remains low. Co-design aims to enhance intervention relevance and engagement. This systematic review evaluated cognition outcomes following physical activity interventions incorporating co-design and determined whether cerebrovascular function was assessed. Six databases were searched until 28 August 2026 in accordance with Preferred Reporting Items for Systematic Reviews and Meta-Analyses guidelines and PROSPERO registration (CRD420261279510). Eligible studies investigated cognition or cerebrovascular function following co-designed physical activity interventions in adults. Methodological quality was assessed using the Joanna Briggs Institute Critical Appraisal Tool, and findings were synthesised narratively. Of 757 records identified, four randomised controlled trials involving 315 participants were included. Co-design approaches varied, and adherence was favourable in three studies but lower in the refugee camp-based intervention. Global cognition and memory showed the most promising findings, although statistically significant intervention effects were limited to one study. Executive function, attention and working memory showed no consistent changes. No study assessed cerebrovascular function. Physical activity interventions incorporating co-design produced promising but inconsistent cognitive outcomes, but the independent contribution of co-design could not be determined. Future trials should integrate cerebrovascular function and cognition assessments to clarify mechanisms and determine whether co-design provides benefits beyond conventional intervention development.

## 1. Introduction

Dementia represents a major and growing public health challenge globally and in Australia [1]. Globally, dementia is a leading cause of disability and mortality in older adults and contributes substantially to loss of independence and quality of life [2,3]. Over 433,300 Australians are currently living with dementia, with projections suggesting this will increase to 852,000 by 2054 as the population ages [1]. Increasing evidence indicates that cerebrovascular dysfunction plays a central role in the development and progression of dementia, highlighting the importance of targeting modifiable pathways to delay or prevent cognitive decline. Accordingly, there is a critical need for effective prevention strategies that target underlying mechanisms rather than relying solely on disease management.

Cerebrovascular dysfunction is increasingly recognised as a key contributor to the development and progression of dementia. Reduced cerebrovascular function, leading to reduced cerebral blood flow, is a characteristic feature of dementia and may precede any measurable decline in cognition [4,5,6]. The brain is a highly metabolically active organ that relies on tightly regulated cerebral blood flow to meet metabolic demands [7,8]. Cerebrovascular function reflects the capacity of the cerebrovasculature to regulate cerebral blood flow through mechanisms including cerebral autoregulation and neurovascular coupling, both of which depend on endothelial integrity and cerebrovascular responsiveness [5,9]. With ageing, endothelial dysfunction, oxidative stress, arterial stiffness and reduced nitric oxide bioavailability impair these processes, resulting in reduced cerebral perfusion and compromised cognition [10,11]. These vascular changes limit the ability of the cerebrovasculature to meet neuronal metabolic demand and contribute to the development of cognitive impairment and dementia [5].

While advancing age remains the primary risk factor for dementia, a substantial proportion of cases are attributable to modifiable risk factors. Cardiometabolic and lifestyle-related factors, including physical inactivity, obesity, hypertension and low education are strongly associated with increased dementia risk [12,13]. These factors contribute to endothelial dysfunction and cardiovascular disease, which further compromise cerebrovascular function and increase the likelihood of cognitive impairment [1,11,14,15]. It is estimated that modifiable risk factors may account for approximately 40% of dementia cases worldwide, highlighting the importance of prevention strategies that target vascular and lifestyle pathways [16]. Among these, physical inactivity represents a central and modifiable contributor to both cerebrovascular dysfunction and cognitive decline.

Physical activity and structured exercise training are well-established non-pharmacological interventions for improving cerebrovascular function and cognition [17]. Aerobic exercise training has been shown to enhance cerebral blood flow, improve cerebrovascular function and increase neuroplasticity, leading to improvements in cognition [17,18]. Interventions consisting of moderate intensity aerobic exercise performed three to four times per week over 12 weeks have demonstrated reductions in arterial stiffness and improvements in cerebral haemodynamics and vasomotor reactivity [19,20,21]. Similarly, resistance training interventions have been associated with improvements in cognition following moderate to high intensity programs lasting from 24 weeks to nine months [22,23,24]. These benefits are proposed to be mediated through improvements in cerebrovascular function, including increased cerebral blood flow, reduced inflammation and enhanced endothelial function [17]. Despite this evidence, the translation of physical activity interventions into real-world settings remains limited.

Barriers to participation and adherence represent a major limitation to the effectiveness of physical activity interventions in older adults. Common perceived barriers include fear of injury, poor health, low confidence and reduced energy levels, while extrinsic barriers include lack of transport, limited access to facilities, scheduling conflicts and financial constraints [25,26]. These barriers are particularly pronounced in regional, rural and remote populations, where access to services is limited and health outcomes are poorer compared with metropolitan areas [27]. As a result, participation in physical activity remains low. In Australia, more than 70% of older adults do not meet recommended levels of activity, and only 13% achieve both aerobic and resistance training guidelines [28]. This disparity highlights a clear gap between the efficacy of physical activity interventions and their real-world implementation in populations most at risk of cognitive decline.

Co-design has emerged as a strategy to address these implementation challenges by involving end users and stakeholders in the development of health interventions. Co-design is a participatory approach that promotes shared ownership by engaging individuals in identifying barriers, designing solutions and shaping intervention delivery [29,30]. It aims to enhance relevance, feasibility and engagement by aligning interventions with the needs, preferences and lived experiences of target populations [31]. Evidence suggests that co-designed physical activity interventions are associated with improved acceptability and engagement in older adults and may enhance participation through improved alignment with individual and community needs [31]. However, existing literature has primarily focused on feasibility, process outcomes and self-reported measures, with limited assessment of objective physiological outcomes.

Despite increasing adoption of co-design in research and its growing requirement in funding frameworks, there remains a lack of evidence evaluating its impact on clinical outcomes. Current co-designed physical activity interventions in older adults have largely focused on physical health, mobility and social outcomes, with few studies assessing cerebrovascular function or using standardised measures of cognition [31]. Further, variability in the definition, implementation and reporting of co-design processes limits the ability to determine its efficacy. To date, no systematic review has synthesised the evidence examining whether co-designed physical activity interventions improve both cerebrovascular function and cognition. As such, it remains unclear whether co-design improves not only engagement and adherence but also the physiological outcomes that underpin dementia prevention. Although ageing and dementia provide important clinical contexts for this review, physical activity interventions incorporating co-design may be applied across diverse adult populations in whom cognition is clinically relevant. The review therefore retained a broad adult population to capture the range of clinical and non-clinical contexts in which these interventions have been evaluated. Therefore, the aim of this systematic review was to evaluate cognitive outcomes following physical activity interventions incorporating co-design and determine whether cerebrovascular function was assessed.

## 2. Materials and Methods

The protocol for this systematic review was registered with the International Prospective Register of Systematic Reviews (PROSPERO; CRD420261279510). The Peer Review of Electronic Search Strategies (PRESS) 2015 Guideline Statement was used to inform the development of the search strategy, and reporting was conducted in accordance with the Preferred Reporting Items for Systematic Reviews and Meta-Analyses (PRISMA) guidelines [32,33]. A systematic search of six electronic databases, including PubMed, MEDLINE, Scopus, Web of Science, CINAHL, and OVID (EMBASE), was undertaken to identify studies examining the effects of co-designed physical activity interventions on cerebrovascular function and cognition in adult humans up until 6 March 2026. The complete database searches were rerun on 28 August 2026 before manuscript submission to identify any newly published eligible studies. No additional eligible studies were identified. The search terms used across the databases consisted of keywords including “co-design”, “co?design”, “co-create”, “co?create”, “particip* design”, “stakeholder engagement”, “user-cent?ed design”, “consumer design”, “co-produc*”, “patient?cent*d”, “physical activity”, “exercise”, “exercise*”, “resistance training”, “muscle strength training”, “concurrent exercise”, and “aerobic*”, in combination with terms relating to cerebrovascular function and cognition including “cerebrovascular circulation”, “cerebral blood flow”, “cerebral blood volume”, “cerebral perfusion”, “cerebral blood velocity”, “cerebrovascular function”, “cognition”, “executive function”, “attention”, “decision-making”, “memory”, “processing speed”, and “cogniti*”, as well as medical subject headings (MeSH) terms, index terms and subject headings relevant to each database. Adaptations to the strategy were made as required for databases without MeSH indexing to maintain consistency in scope and relevance. The complete PubMed search strategy is presented in Table 1, with the exact final search strategies for all six databases, including the initial and updated search dates, provided in Supplementary Table S1.

Studies were eligible for inclusion if they were peer-reviewed, written in English, had full-text availability, and involved human participants aged 18 years or older. The broad adult criterion was selected to identify relevant interventions across clinical and non-clinical populations rather than restrict the review to ageing or dementia contexts. Studies were required to report outcomes relating to cerebrovascular function and/or cognition following co-designed physical activity interventions. For the purposes of this review, co-design was operationalised as the active involvement of members of the target population or relevant stakeholders in developing, adapting or individually tailoring the content or delivery of the physical activity intervention [29,31]. To meet the minimum criterion for inclusion, stakeholder involvement was required to extend beyond intervention participation or research data collection and to have an identifiable influence on intervention development, adaptation or delivery. Eligible approaches included participant testing and refinement of intervention activities, collaborative goal setting and intervention planning, stakeholder consultation, or iterative development and usability testing. Co-design was therefore treated as an eligibility characteristic of the intervention rather than an independently evaluated intervention component. Randomised and non-randomised primary intervention studies were eligible for inclusion. Unpublished articles, grey literature, reviews, abstracts, conference presentations, and studies involving participants younger than 18 years were excluded.

Following completion of database searches, duplicate studies were removed, with all remaining studies imported into Joanna Briggs Institute (JBI) Sumari, where they were subsequently screened by title and abstract by two researchers (AS and ESB). A third researcher (DEM) resolved any conflicts. Following this, two researchers (AS and ESB) completed full-text screening, with a third reviewer (DEM) resolving any conflicts that arose. Reference lists of included studies were also screened to identify additional eligible studies.

The methodological quality and risk of bias of included studies were assessed using the JBI Critical Appraisal Tool for Randomised Controlled Trials [34]. This tool evaluates domains including randomisation, allocation concealment, baseline comparability, blinding of participants, personnel and outcome assessors, treatment group comparability, completeness of follow-up, intention-to-treat analysis, consistency and reliability of outcome measurement, and appropriateness of statistical analysis. Quality assessment was conducted independently by two reviewers (AS and ESB), with discrepancies resolved through discussion and by a third researcher (DEM). Studies scoring less than 50% positive responses were categorised as low quality and excluded, those scoring between 50% and 75% were considered moderate quality, and those scoring greater than 75% were considered high quality [34].

Data extraction was undertaken independently by two reviewers (AS and ESB) using a pre-specified extraction framework in Microsoft Excel. Extracted data included study design and participant characteristics, intervention details, including description of the co-design process, exercise modality, frequency, intensity and duration, comparator conditions where applicable, cerebrovascular and cognitive outcomes, methods of outcome measurement, and main findings. Where required, Supplementary Materials were consulted to ensure completeness of extracted data. All extracted data were cross-checked for accuracy prior to synthesis, with unresolved discrepancies adjudicated by a third researcher (DEM).

Due to substantial heterogeneity across included studies, it was not possible to conduct a meta-analysis. Therefore, a structured narrative synthesis was undertaken. Heterogeneity was evident in study design, participant populations, intervention characteristics, including variations in the degree and implementation of co-design, exercise type and dosing regimens, intervention duration, comparator conditions, and outcome measures used to assess cognition and cerebrovascular function. Cognitive outcomes varied across domains and assessment tools, while no eligible studies reported measures of cerebrovascular function. Findings from the included studies were synthesised narratively in accordance with recommended guidelines [35]. The direction, magnitude and consistency of effects were considered alongside statistical significance, with effect-size estimates reported where available.

## 3. Results

**Figure 1:**
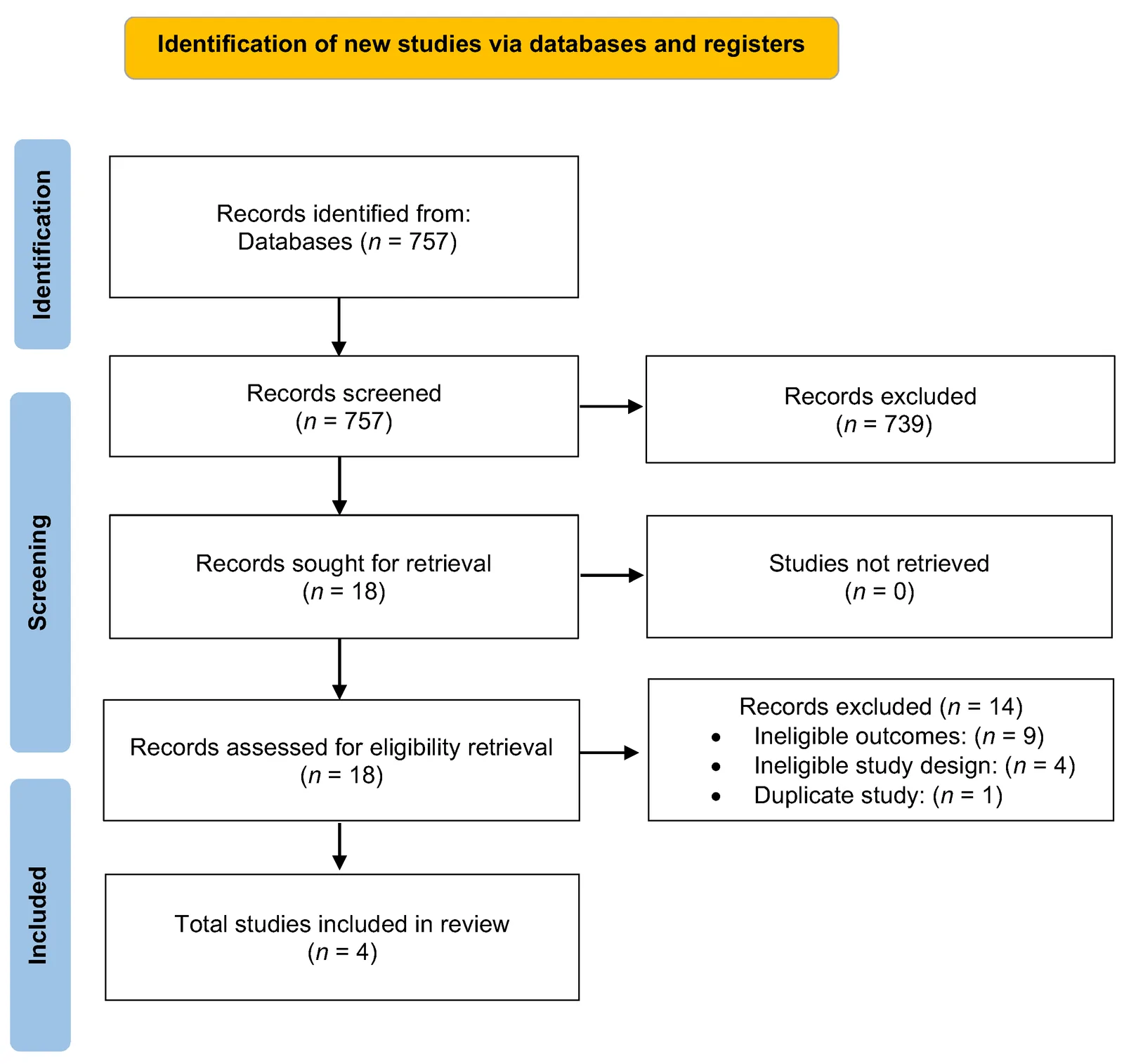
PRISMA flow diagram outlining the screening process and outcomes at each stage.

The PRISMA flow diagram outlining the study selection process is presented in Figure 1. A total of 757 records were identified from database searches. Following this, all 757 records were screened by title and abstract, with 739 records excluded due to irrelevance or failure to meet the inclusion criteria. Subsequently, 18 studies were sought for full-text retrieval, and all were successfully retrieved. Full-text assessment resulted in the exclusion of 14 studies for the following reasons: ineligible outcomes (*n* = 9), ineligible study design (*n* = 4), and duplicate study (*n* = 1). In total, four randomised controlled trials including a total of 315 participants met the inclusion criteria and were included in the final synthesis [36,37,38,39]. The methodological quality and risk of bias of the four included studies are presented in Table 2. The characteristics and outcomes of the included studies are presented in Table 3 and are described narratively below. The results described in Table 3 were reported to be statistically significant (*p* < 0.05), while no change indicates the absence of a statistically significant effect.

**Table 2:** Literature quality assessment of included studies via the JBI Critical Appraisal Tool.

Citation	Q1	Q2	Q3	Q4	Q5	Q6	Q7	Q8	Q9	Q10	Q11	Q12	Q13	Study Quality	Include (Y/N)
[36]	Y	N	Y	N	N	Y	Y	Y	Y	Y	Y	Y	Y	High	Y
[37]	Y	Y	Y	N	N	Y	Y	Y	Y	Y	Y	Y	Y	High	Y
[38]	Y	N	Y	N	N	N	Y	Y	Y	Y	Y	Y	Y	Moderate	Y
[39]	Y	N	Y	N	N	Y	Y	Y	Y	Y	Y	Y	Y	High	Y
Abbreviations: Y: Yes; N: No; U: Unclear; N/A: Not applicable.															
Randomised Controlled Trial Study Appraisal Questions: Q1: Was true randomisation used for assignment of participants to treatment groups? Q2: Was allocation to treatment groups concealed? Q3: Were treatment groups similar at baseline? Q4: Were participants blind to treatment assignment? Q5: Were those delivering treatment blind to treatment assignment? Q6: Were outcome assessors blind to treatment assignment? Q7: Were treatment groups treated identically other than the intervention of interest? Q8: Was follow-up complete and, if not, were differences between groups in terms of their follow-up adequately described and analysed? Q9: Were participants analysed in the groups to which they were randomised? Q10: Were outcomes measured in the same way for treatment groups? Q11: Were outcomes measured in a reliable way? Q12: Was appropriate statistical analysis used? Q13: Was the trial design appropriate and were any deviations from the standard RCT design accounted for in the conduct and analysis of the trial?															

Green represents a positive response and inclusion in the study. Orange represents a negative response.

**Table 3:** Characteristics of co-designed physical activity interventions and cognitive outcomes.

Reference	Study Design	Participant Information	Intervention Details	Cognitive Tests	Cognitive Domains Assessed	Effect on Cognition	Secondary Outcomes
[36]	Randomised controlled feasibility trial	Community-dwelling older adults aged 65 years and above Control (71 ± 6 years; *n* = 26) Intervention (72 ± 5 years; *n* = 50)	Co-design: Nine target-population members trialled and refined the intervention activities Intervention: Online dual-task training combining cognition activities with chair-based physical activity Duration: 12 weeks, 60 min twice weekly Comparator: Eight online interactive health education sessions	Montreal Cognitive Assessment Digit Span test Memory Inventory in Chinese Stroop test	Global cognition Attention/working memory Working memory Executive function	↔ ↔ ↔ ↔	↑ Wellbeing ↔ Physical function
[37]	Randomised controlled pilot trial	Community-dwelling older adults with dementia Control (79 ± 7 years; *n* = 29) Intervention (78 ± 6 years; *n* = 31)	Co-design: Participants with dementia, caregivers and clinical staff established individual rehabilitation goals and tailored intervention plans Intervention: Multidisciplinary rehabilitation including group exercise and social interaction Duration: 20-week rehabilitation programme, including 45 min exercise sessions twice weekly for 16 weeks Comparator: Usual care	Mini-Mental State Examination Verbal fluency test	Global cognition Executive function	↔ ↔	↔ Physical function ↔ Behavioural outcomes
[38]	Randomised controlled trial	Forcibly displaced individuals Control (29 years; *n* = 68) Intervention (29 years; *n* = 74)	Co-design: Service users, staff and camp management informed intervention activities, delivery and strategies addressing participation barriers Intervention: Group-based fitness training, martial arts, ball sports and dance Duration: 10 weeks, 50 to 60 min per session, with activities available up to five days per week Comparator: Waitlist control	Flanker test Oddball task	Inhibitory control Attention	↔ ↔	↑ Cardiorespiratory fitness
[39]	Randomised controlled trial	Individuals with mild neurocognitive disorder Control (72 ± 10 years; *n* = 17) Intervention (74 ± 8 years; *n* = 20)	Co-design: People with mild neurocognitive disorder, health professionals, researchers and industry experts iteratively tested and refined the intervention Intervention: Home-based Brain-IT exergame training combining motor-cognition activities with heart rate variability biofeedback Duration: 12 weeks, at least 24 min per session and five sessions per week Comparator: Usual care	Quick Mild Cognitive Impairment screen WMS-IV-LM, immediate recall WMS-IV-LM, delayed recall WMS-IV-LM, recognition Digit Span Forward Trail Making Test, Part A TAP Go/No-Go HOTAP picture-sorting test, Part A Digit Span Backward Trail Making Test, Part B Mental Rotation Task	Global cognition Immediate verbal memory Delayed verbal memory Recognition memory Attention and immediate memory Complex attention Complex attention and response inhibition Executive function and planning Executive function and working memory Executive function and cognitive flexibility Visuospatial cognition	↑ ↑ ↑ ↔ ↔ ↔ ↔ ↔ ↔ ↔ ↔	↔ Heart rate variability ↔ Quality of life

Abbreviations: HOTAP, picture-sorting test of everyday action planning; TAP, Test of Attentional Performance; WMS-IV-LM, Wechsler Memory Scale, Fourth Edition, Logical Memory subtest. ↑ indicates a statistically significant improvement; ↓ indicates a statistically significant reduction; ↔ indicates no statistically significant intervention effect. Table 3 provides a concise summary of statistically significant intervention effects, while the narrative synthesis reports the direction, magnitude and consistency of findings, including effect-size estimates where available.

Across the four included studies, methodological quality was rated as moderate to high. Three studies were classified as high quality [36,37,39], demonstrating strengths in randomisation, outcome measurement, follow-up, and statistical analysis. The remaining study was classified as moderate quality [38], with limitations most commonly relating to allocation concealment and the absence of blinding of participants and intervention personnel. Blinding of participants and those delivering the intervention was not implemented in any study. However, outcome assessor blinding was reported in most studies, supporting the reliability of outcome assessment. All studies used consistent outcome measurement approaches across groups and applied appropriate statistical analyses. No studies were rated as low quality, and all included studies were retained in the final synthesis.

### 3.1. Study Characteristics

All included studies utilised randomised controlled trial designs and were conducted across heterogeneous populations and settings [36,37,38,39]. Sample sizes across studies ranged from 37 to 142 participants. Chau et al. (2025) recruited 76 community-dwelling older adults in Hong Kong (intervention *n* = 50, control *n* = 26), with a mean age of 72 ± 5 years and 64% female representation in the intervention group [36]. Participants were apparently cognitively healthy and aged ≥65 years. Hasselgren et al. (2024) included 60 community-dwelling older adults with dementia (intervention *n* = 31, control *n* = 29), with a mean age of 79 ± 6 years and 58% female participants [37]. This sample represented a clinically impaired population with established cognitive decline. Manser and de Bruin (2024) analysed 37 participants with mild neurocognitive disorder (intervention *n* = 20, control *n* = 17), with a mean age of 73 ± 9 years [39]. This cohort represented an early-stage clinical population at risk of progression to dementia. In contrast, Knappe et al. (2024) included 142 forcibly displaced individuals (53% female) residing in a refugee camp, with a substantially younger mean age of 29 years (95% CI: 27.7–30.8) [38]. This study differed markedly from the others in both age and sociodemographic context, representing a non-clinical, high-stress population at risk of post-traumatic stress disorder.

### 3.2. Co-Design Methodology

Co-design approaches varied across the included studies. Chau et al. (2025) involved nine members of the target population in trialling potential dual-task activities, with their feedback informing the selection and refinement of the final intervention components [36]. Hasselgren et al. (2024) used a person-centred rehabilitation process in which participants with dementia, caregivers and clinical staff established individual rehabilitation goals and tailored intervention plans [37]. Knappe et al. (2024) involved service users, staff and camp management through preliminary consultation, informal discussions and ongoing debriefing, with feedback informing activity selection, session content, timing, location and strategies to address participation barriers [38]. Manser and de Bruin (2024) used an iterative development process involving people with mild neurocognitive disorder, health professionals, researchers and industry experts to establish design requirements and refine the intervention through repeated feasibility, usability, safety and acceptance testing [39].

Intervention duration and structure varied from 10 to 20 weeks across studies. Chau et al. (2025) and Manser and de Bruin (2024) both delivered 12-week interventions. Chau et al. (2025) implemented an online dual-task training programme delivered twice weekly for 60 min sessions, while Manser and de Bruin (2024) delivered a home-based exergame intervention with a prescribed minimum frequency of five sessions per week and ≥120 min of total weekly training [36,39]. Hasselgren et al. (2024) employed a 20-week multidisciplinary rehabilitation programme that included 45 min supervised exercise and social interaction sessions delivered twice weekly for 16 weeks, with follow-up assessments extending to 36 months [37]. In comparison, Knappe et al. (2024) implemented a 10-week co-designed exercise and sport intervention consisting of 50–60 min sessions delivered up to five days per week [38].

Interventions encompassed diverse physical activity modalities. Chau et al. (2025) utilised online dual-task training combining cognitive exercises with chair-based physical activity [36]. Hasselgren et al. (2024) delivered a person-centred rehabilitation programme incorporating high-intensity functional exercise and interdisciplinary care components [37]. Manser and de Bruin (2024) employed a technology-based “Brain-IT” intervention integrating exergaming, motor–cognitive training, and heart-rate variability biofeedback [39]. Knappe et al. (2024) implemented a co-designed programme including fitness training, martial arts, ball sports, and dance activities tailored to participant preferences [38]. Comparator conditions also varied across studies and included online interactive health education [36], usual care [37,39], and a waitlist control condition [38]. Across studies, interventions frequently integrated physical and cognitive components, although substantial heterogeneity was evident in participant populations, delivery formats, intervention intensity, and contextual factors. However, despite inclusion of four eligible studies, none reported direct measures of cerebrovascular function.

### 3.3. Cognitive Outcomes Following Physical Activity Interventions Incorporating Co-Design

All included studies evaluated cognitive outcomes, although measures and domains varied considerably. Chau et al. (2025) reported improvements in subjective memory complaints and cognition following a 12-week online dual-task intervention [36]. Within-group analyses demonstrated moderate-to-large effect sizes for subjective memory improvement at mid-intervention (*d* = 0.65), post-intervention (*d* = 0.43), and follow-up (*d* = 0.85). Between-group differences in global cognition favoured the intervention, although effect sizes were small to moderate (*d* = 0.34 at mid-intervention; *d* = 0.23 post-intervention). However, the intervention did not outperform the attention control condition for subjective memory, and findings for working memory and executive function were inconsistent.

Manser and de Bruin (2024) observed significant improvements in global cognition following the “Brain-IT” intervention, with a large effect size (*p* = 0.007, η^2^*p* = 0.197) [39]. Secondary outcomes also demonstrated significant improvements in immediate and delayed verbal recall, both with large effect sizes (η^2^*p* = 0.154 and 0.204, respectively). Furthermore, 55% of participants in the intervention group achieved clinically meaningful improvement, compared with 23% in the control group. In contrast, Hasselgren et al. (2024) reported no significant between-group differences in global cognition or other cognitive outcomes over the 36-month follow-up period, despite some trends favouring the intervention [37]. This suggests limited cognitive benefit from the multidimensional rehabilitation programme in this population with established dementia. Finally, Knappe et al. (2024) assessed sustained attention and inhibitory control using the Oddball paradigm and Flanker task, respectively. No direct intervention effects were observed for reaction time or accuracy. However, the intervention significantly improved cardiorespiratory fitness, and improved cardiorespiratory fitness was associated with faster reaction times across the cognition tasks [38].

### 3.4. Psychological and Mental Health Outcomes Following Physical Activity Interventions Incorporating Co-Design

Psychological outcomes varied depending on study population and intervention type. Knappe et al. (2024) assessed post-traumatic stress disorder symptom severity and included it as a covariate in the cognition and pain analyses. However, the effect of the intervention on post-traumatic stress disorder symptoms was not reported in this secondary analysis [38]. Chau et al. (2025) reported qualitative and quantitative improvements in psychosocial wellbeing, with participants describing enhanced mood, enjoyment, and social interaction [36]. However, changes in depressive symptoms were inconsistent and not clearly superior to the control condition. Manser and de Bruin (2024) assessed psychosocial outcomes including quality of life, depression, anxiety, and stress [39]. While a moderate improvement in quality of life was observed (approaching significance, *p* = 0.065), other psychological outcomes did not demonstrate consistent or statistically significant effects. Hasselgren et al. (2024) reported no significant differences in neuropsychiatric symptoms between intervention and control groups over time, despite minor trends favouring the intervention [37].

### 3.5. Physical and Functional Outcomes Following Physical Activity Interventions Incorporating Co-Design

Physical and functional outcomes were primarily assessed in studies involving older adults. Hasselgren et al. (2024) demonstrated trends toward improved gait speed, physical activity, and neuropsychiatric outcomes; however, these did not reach statistical significance [37]. Chau et al. (2025) included functional measures such as activities of daily living and physical performance, although no consistent improvements were observed across these domains [36]. Manser and de Bruin (2024) assessed gait and functional outcomes but reported largely inconclusive findings, with no consistent improvements across most physical domains [39]. In contrast, Knappe et al. (2024) incorporated structured sport and exercise participation, which contributed to improved overall engagement in physical activity, although formal functional measures were not the primary focus [38].

### 3.6. Adherence and Feasibility

Three of the four studies reported high levels of adherence and feasibility, suggesting that co-designed physical activity interventions were generally well tolerated across populations. Chau et al. (2025) reported that 72% of participants in the intervention group attended at least 75% of sessions, exceeding predefined feasibility thresholds [36]. Hasselgren et al. (2024) observed an attendance rate of approximately 75% for exercise sessions, with no serious adverse events linked to the intervention [37]. Manser and de Bruin (2024) reported high adherence rates, with participants completing an average of 90% of prescribed sessions and training durations [39]. In Knappe et al. (2024), approximately 41% of intervention participants completed the recommended two sessions per week, while approximately 33% participated once per week [38]. This lower adherence likely reflected the unstable refugee camp setting, participant movement out of the camp, and the flexible, self-selected structure of the physical activity programme. These findings describe adherence and feasibility within co-designed interventions but do not establish that co-design itself improved engagement or adherence.

## 4. Discussion

The primary objective of this systematic review was to evaluate cognitive outcomes following physical activity interventions incorporating co-design and determine whether cerebrovascular function was assessed. Synthesis of the four included randomised controlled trials revealed mixed cognitive outcomes. Global cognition and memory produced the most favourable findings, but statistically significant intervention effects were demonstrated in only one included study. Conversely, executive function, attention, and working memory did not demonstrate consistent changes across studies. A key finding of this review is that none of the included studies assessed cerebrovascular function. Collectively, physical activity interventions incorporating co-design produced promising but inconsistent cognitive outcomes. Importantly, the absence of cerebrovascular measures prevents identification of the mechanisms underlying observed changes in cognition.

The promising findings for memory and global cognition may be attributable to the combined physical and cognitive demands embedded within the physical activity interventions. Interventions incorporating high task complexity and concurrent cognitive engagement, such as dual-task training and exergaming, require substantial cognitive processing in addition to physical exertion [39]. This combination is proposed to enhance neuroplasticity through exercise-induced upregulation of neurotrophic factors and task-specific cognitive stimulation, contributing to structural and functional neural adaptation [40,41].

Intervention duration and the inclusion of an explicit cognitive training component may also have influenced the observed outcomes. Recent meta-analytical evidence suggests that shorter intervention durations can produce measurable improvements in memory and global cognition [42]. The online group-based dual-task training investigated Chau et al. (2025) and the Brain-IT exergame with biofeedback breathing evaluated by Manser and de Bruin (2024) both integrated explicit cognitive training components and produced more favourable findings than interventions focused predominantly on physical activity [36,39]. Notably, Manser and de Bruin (2024) reported statistically significant improvements with large effect sizes in global cognition and both immediate and delayed verbal recall, supporting the premise that combined physical and cognitive stimuli may act synergistically to preserve and enhance cognition [39]. These findings should be interpreted cautiously because they arose from different populations, intervention packages and cognition assessments, rather than from direct comparisons of cognition domains within a common intervention context.

Heterogeneity in cognitive outcomes across the included studies is likely explained by several methodological differences. Participant populations varied substantially, ranging from apparently cognitively healthy older adults to individuals with mild neurocognitive disorder, established dementia, and younger forcibly displaced individuals at high risk of post-traumatic stress disorder. These differences in baseline cognitive status likely contribute to variation in adaptive capacity and response to intervention, as cognitive decline and neurodegenerative progression are associated with reductions in neuroplastic potential and responsiveness to therapeutic interventions [43]. Additionally, there was considerable variation in intervention protocols, including physical activity modality (e.g., high-intensity functional training, exergaming, chair-based exercise), duration (10 to 20 weeks), and training frequency and intensity. Such variability suggests that some interventions may not have reached the minimum dose threshold required to elicit meaningful neuroadaptation [40]. Furthermore, several studies were conducted as pilot or feasibility trials with small sample sizes, limiting statistical power and increasing variability in outcomes. Accordingly, the included studies should not be interpreted as representing a clinically homogeneous evidence base or as estimating a common intervention effect across populations.

Findings from this review also suggest that the clinical status of the target population is a critical determinant of intervention efficacy. Individuals with established dementia, as investigated by Hasselgren et al. (2024), demonstrated minimal cognitive improvement following intervention [37]. This limited responsiveness may reflect reduced neuroplastic capacity associated with advanced neurodegenerative processes, where structural and functional brain changes may be less amenable to modification [43]. These findings highlight the importance of intervention timing. Co-designed physical activity interventions are unlikely to reverse advanced neurodegeneration. However, they may be more effective when implemented as preventative strategies in early-stage or non-clinical populations [17,43]. Individuals with mild neurocognitive impairment or those at elevated risk of cognitive decline may retain sufficient neuroplastic potential to benefit from targeted interventions, supporting a shift towards earlier intervention in clinical practice [17,43].

The absence of cerebrovascular outcomes represents the most significant gap identified within the current evidence base. Cerebrovascular function, through its regulation of cerebral blood flow, is widely recognised as a central mechanistic link between physical activity and cognition [5,17,44]. The lack of these measures severely restricts interpretation of the underlying mechanisms driving observed cognitive changes. Without direct assessment of cerebrovascular function, it is not possible to determine whether improvements in cognition were mediated by central adaptations within the cerebrovasculature, systemic cardiovascular changes, or whether they reflect behavioural, psychological, or task-specific learning effects. The absence of cerebrovascular and related physiological data prevents the development of mechanistic models linking physical activity exposure to systemic and central adaptations, limiting the ability to determine how these interventions influence cognition. As a result, clinicians are left without objective measures to guide exercise prescription or to monitor cerebrovascular or other physiological adaptations relevant to brain health. Current evidence therefore demonstrates potential cognitive benefit without explaining the pathways through which these effects occur.

Cerebrovascular function assessments relevant to cognition include resting cerebral blood flow or cerebral perfusion, cerebrovascular responsiveness to hypercapnia, and neurovascular coupling assessed through the response of the cerebrovasculature to cognitive stimuli [5,9,10,17,44]. Resting cerebral blood flow reflects the cerebral blood supply available to support neuronal function, while cerebrovascular responsiveness to hypercapnia assesses the capacity of the cerebrovasculature to dilate in response to a physiological stimulus. Neurovascular coupling may be particularly relevant to cognition because it reflects the capacity of the cerebrovasculature to increase cerebral blood flow in response to cognitive activation and increased neuronal metabolism. In sedentary, obese older adults, 16 weeks of aerobic exercise training improved cerebrovascular responsiveness to hypercapnia, cerebrovascular responsiveness to cognitive stimuli and composite cognition, with exercise-session completion strongly associated with improved cerebrovascular responsiveness to cognitive stimuli [45]. However, whether improvements in these assessments following exercise training directly correlate with improvements in cognition remains unclear, reinforcing the need for future trials to assess these outcomes concurrently and examine their relationships.

Although co-design was a requirement for inclusion in this review, the approaches used varied considerably across studies. Co-design approaches differed in the stakeholders involved, the methods used, the stage at which engagement occurred, and the aspects of intervention development or delivery that were influenced. These approaches ranged from participant trialling and refinement of intervention activities to individual goal setting, stakeholder consultation, and iterative development involving end users, health professionals, researchers, and industry representatives. This variability is consistent with broader evidence demonstrating inconsistency in the definition, implementation, and reporting of co-design across health and physical activity research [29,31]. Although differences in co-design may have influenced intervention relevance, delivery, and engagement, the inclusion of only four heterogeneous studies prevents meaningful comparison of the approaches or determination of whether one method was more effective than another. Greater use of standardised co-design frameworks and consistent reporting of stakeholder involvement, engagement methods, and resulting intervention changes is needed to support comparison across future studies [29,31].

Adherence and feasibility were favourable in three studies but lower in Knappe et al. (2024), where the intervention was delivered within an unstable refugee camp setting [38]. These findings suggest that co-design may support engagement and acceptability by involving end users and relevant stakeholders in tailoring intervention delivery and addressing barriers to participation [29,31]. However, conventional physical activity and exercise training interventions have also demonstrated benefits for cognition and cerebrovascular function [17,42], and the current evidence does not establish whether co-design provides additional benefits beyond conventional researcher-led intervention development. Indirect comparison with the broader physical activity literature is limited by differences in participant populations, intervention characteristics, comparator conditions, and outcome assessments. It is therefore not possible to determine whether co-design is more or less effective than conventional intervention development, and any recommendation to favour one approach would currently be unsupported by comparative evidence. Further primary research directly comparing intervention development approaches is required before the additional effects of co-design on adherence, cognition, or cerebrovascular function can be established.

When considered alongside the broader physical activity literature, the findings of this review provide several potential implications for exercise professionals and researchers. Structured and progressive exercise programmes may offer advantages over general or unstructured physical activity advice, likely due to greater control over intensity, volume, and cognitive demand [24]. The broader evidence also supports multimodal interventions that integrate both physical and cognitive components. Dual-task training paradigms may be beneficial because they simultaneously challenge motor and cognitive systems [41]. Importantly, interventions must also adhere to the principle of sufficient training dose. Without adequate intensity, duration, and progression, exercise interventions are unlikely to reach the threshold required to induce meaningful neuroplastic or cerebrovascular changes to enhance cognition [17]. Finally, intervention prescription should be targeted toward populations most likely to benefit, particularly individuals in early stages of cognitive decline or those at elevated risk, where the potential for adaptation remains greatest [43].

### Strengths, Limitations and Future Directions

This systematic review has several key strengths. It provides a focused evaluation of cognitive outcomes following physical activity interventions incorporating co-design, an emerging approach within personalised and translational health research. Although both randomised and non-randomised intervention studies were eligible, all included studies were randomised controlled trials and were rated as moderate or high quality. The use of a predefined and systematic methodology further supports the transparency and reproducibility of the findings. However, several limitations should be considered. The evidence base was limited, with only four eligible studies identified, reflecting a scarcity of research in this area. Substantial heterogeneity was observed across participant populations, co-design approaches, intervention designs, comparator conditions and outcome measures, limiting comparability between studies. This clinical diversity also limits the generalisability of the findings to any single population, including older adults, people with cognitive impairment or younger adults exposed to substantial psychosocial stress. Cognition assessments varied considerably, reducing consistency in outcome reporting. In addition, sample sizes were generally small, which may have limited statistical power and contributed to variability in findings. None of the included studies assessed cerebrovascular function. This represents a critical limitation of the current evidence base, as it prevents determination of the mechanisms underlying observed cognitive changes. Without direct assessment of cerebrovascular function, it remains unclear whether cognition outcomes were influenced by central cerebrovascular adaptations, systemic physiological changes, task-specific learning effects or a combination of these factors. This limits both mechanistic interpretation and clinical translation.

Future research should therefore prioritise the inclusion of cognition and cerebrovascular function within the same study design to better establish mechanistic pathways. Larger, adequately powered studies are needed to provide more precise estimates of intervention effects and examine whether these effects are sustained. Greater consistency in cognition assessments and reporting of co-design approaches would also improve comparison across studies. Further research is required to determine whether co-design provides additional benefits for engagement, adherence or cognition beyond conventional researcher-led intervention development. Future studies should also examine populations at different stages of cognitive decline to determine whether clinical status influences responsiveness to intervention.

## 5. Conclusions

In conclusion, cognitive outcomes following physical activity interventions incorporating co-design are promising but insufficient to determine whether these interventions consistently improve cognition. The most favourable findings were observed for global cognition and memory, although statistically significant intervention effects were limited to one study. The independent contribution of co-design could not be determined because no included trial compared an intervention incorporating co-design with an otherwise equivalent conventionally developed intervention. The current evidence base is limited and heterogeneous. Substantial differences in age, baseline cognition, clinical context and intervention characteristics also prevent broad generalisation across adult populations. The absence of cerebrovascular function assessments across all included trials represents a critical gap, preventing identification of the mechanisms underlying observed cognitive changes. Without direct evaluation of cerebrovascular function, it remains unclear whether improvements in cognition are driven by the cerebrovasculature, systemic effects, task-specific responses or a combination of these factors.

Future randomised controlled trials must address this limitation by integrating cerebrovascular function and cognitive measures within the same study design. Establishing these mechanistic pathways and determining the additional contribution of co-design are essential to inform the development and implementation of physical activity interventions for individuals at risk of cognitive decline.

## Figures and Tables

**Table 1:** Search terms used in PubMed.

Search Term #1	Search Term #2	Search Term #3	Search Term #4
“Co-design” OR “co?design” OR “co-create” OR “co?create” Or “Particip*design” OR “Stakeholder engagement” OR “User-Cent?ed design” OR “*consumer design” OR “Co-produc*” OR “patient?cent*d”	“Physical Activity” OR “Exercise” [MeSH] OR “Exercise*” OR “Resistance Training” [MeSH] OR “muscle?strength training” Or *Concurrent exercise* OR “Aerobic*”	“Cerebrovascular circulation” [MeSH] OR “Cerebral blood volume” OR “Cerebral blood flow” OR CBF OR “Cerebral perfusion” OR “Cerebrovascular function” OR “Cerebral blood*velocity”	Cognition [MeSH Terms] OR “Decision Making” [MeSH Terms] OR Attention [MeSH Terms] OR “Executive Function” [MeSH Terms] OR Memory [MeSH Terms] OR “Processing Speed” [MeSH Terms] OR cogniti*

Abbreviations: MeSH: medical subject headings.

## Data Availability

No new data were created or analyzed in this study. Data sharing is not applicable to this article.

## Supplementary Materials

The following supporting information can be downloaded at: https://www.mdpi.com/article/10.3390/life16101714/s1. Table S1: Complete database-specific search strategies.
