# Supplementary material for: Cognitive Outcomes Following Physical Activity Interventions Incorporating Co-Design: A Systematic Review of Randomised Controlled Trials
Source: Life (Basel). 2026 Oct 14;16(10):1714. doi: 10.3390/life16101714 (PMC13706219; doi:10.3390/life16101714)
Supplement: Supplementary file 1 [file life-16-01714-s001.zip › life-4566232-supplementary.pdf]

Supplementary Table S1. Complete database-specific search strategies

Pubmed	(("Co-design" OR "co?design" OR "co-create" OR "co?create" Or "Particip*design" OR "Stakeholder engagement" OR "User-Cent?ed design" OR "*consumer design" OR "Co-produc*" OR "patient?cent*d") AND ("Physical Activity" OR "Exercise" [MeSH] OR "Exercise*" OR "Resistance Training"[Mesh] OR "muscle?strength training" Or *Concurrent exercise* OR "Aerobic*")) AND (("Cerebrovascular circulation" [MeSH] OR "Cerebral blood volume" OR "Cerebral blood flow" OR CBF OR "Cerebral perfusion" OR "Cerebrovascular function" OR "Cerebral blood*velocity") OR (Cognition [MeSH Terms] OR "Decision Making" [MeSH Terms] OR Attention [MeSH Terms] OR "Executive Function" [MeSH Terms] OR Memory [MeSH Terms] OR "Processing Speed" [MeSH Terms] OR cogniti*))
MEDLINE	1. ("Co-design" or "co?design" or "co-create" or "co?create" or "Participdesign" or "Stakeholder engagement" or "User-Cent?ed design" or "consumer design" or "Co-produc" or "patient?centd").mp.<br>2. "Physical Activity".mp. or Exercise/ or "Exercise*".mp. or Resistance Training/ or "muscle?strength training".mp. or "Concurrent exercise".mp. or "Aerobic*".mp.<br>3. Cerebrovascular Circulation/ or "Cerebral blood volume".mp. or "Cerebral blood flow".mp. or CBF.mp. or "Cerebral perfusion".mp. or "Cerebrovascular function".mp. or "Cerebral blood*velocity".mp.<br>4. Cognition/ or Decision Making/ or Attention/ or Executive Function/ or Memory/ or "Processing Speed".mp. or cogniti*.mp.<br>5. 3 or 4<br>6. 1 and 2 and 5
Scopus	((Co-design OR co*design OR co-create OR co*create OR Particip*design OR "Stakeholder engagement" OR "User-Cent*ed design" OR "*consumer design" OR Co-produc* OR patient*cent*d) AND ("Physical Activity" OR INDEXTERMS(Exercise) OR Exercise* OR INDEXTERMS("Resistance Training") OR "muscle*strength training" OR "*Concurrent exercise*" OR Aerobic*)) AND ((INDEXTERMS("Cerebrovascular circulation") OR "Cerebral blood volume" OR "Cerebral blood flow" OR CBF OR "Cerebral perfusion" OR "Cerebrovascular function" OR "Cerebral blood*velocity") OR (INDEXTERMS(Cognition) OR INDEXTERMS("Decision Making") OR INDEXTERMS(Attention) OR INDEXTERMS("Executive Function") OR INDEXTERMS(Memory) OR INDEXTERMS("Processing Speed") OR cogniti*))

Web of Science	((Co-design OR co$design OR co-create OR co$create OR Particip*design OR "Stakeholder engagement" OR "User-Cent$ed design" OR "*consumer design" OR Co-produc* OR patient$cent*d) AND ("Physical Activity" OR ALL=Exercise OR Exercise* OR ALL="Resistance Training" OR "muscle$strength training" OR "*Concurrent exercise*" OR Aerobic*)) AND ((ALL="Cerebrovascular circulation" OR "Cerebral blood volume" OR "Cerebral blood flow" OR CBF OR "Cerebral perfusion" OR "Cerebrovascular function" OR "Cerebral blood*velocity") OR (ALL=Cognition OR ALL="Decision Making" OR ALL=Attention OR ALL="Executive Function" OR ALL=Memory OR ALL="Processing Speed" OR cogniti*))
CINAHL	((Co-design OR co#design OR co-create OR co#create OR Particip*design OR "Stakeholder engagement" OR "User-Cent#ed design" OR "*consumer design" OR Co-produc* OR patient#cent*d) AND ("Physical Activity" OR (MH Exercise+) OR Exercise* OR (MH "Resistance Training+") OR "muscle#strength training" OR "*Concurrent exercise*" OR Aerobic*)) AND (((MH "Cerebrovascular circulation+") OR "Cerebral blood volume" OR "Cerebral blood flow" OR CBF OR "Cerebral perfusion" OR "Cerebrovascular function" OR "Cerebral blood*velocity") OR ((MH Cognition+) OR (MH "Decision Making+") OR (MH Attention+) OR (MH "Executive Function+") OR (MH Memory+) OR (MH "Processing Speed+") OR cogniti*))
OVID (EMBASE)	((Co-design OR co?design OR co-create OR co?create OR Particip*design OR "Stakeholder engagement" OR "User-Cent?ed design" OR "*consumer design" OR Co-produc* OR patient?cent*d) AND ("Physical Activity" OR exp Exercise/ OR Exercise* OR exp "Resistance Training"/ OR "muscle?strength training" OR "*Concurrent exercise*" OR Aerobic*)) AND ((exp "Cerebrovascular circulation"/ OR "Cerebral blood volume" OR "Cerebral blood flow" OR CBF OR "Cerebral perfusion" OR "Cerebrovascular function" OR "Cerebral blood*velocity") OR (exp Cognition/ OR exp "Decision Making"/ OR exp Attention/ OR exp "Executive Function"/ OR exp Memory/ OR exp "Processing Speed"/ OR cogniti*))
